# Faecal biomarkers in type 1 diabetes with and without diabetic nephropathy

**DOI:** 10.1038/s41598-021-94747-8

**Published:** 2021-07-26

**Authors:** Signe Abitz Winther, Miia Maininki Mannerla, Marie Frimodt-Møller, Frederik Persson, Tine Willum Hansen, Markku Lehto, Sohvi Hörkkö, Michael Blaut, Carol Forsblom, Per-Henrik Groop, Peter Rossing

**Affiliations:** 1 Steno Diabetes Center Copenhagen, Niels Steensens Vej 2, 2820 Gentofte, Denmark; 2 Novo Nordisk A/S, Måløv, Denmark; 3 Folkhälsan Institute of Genetics, Folkhälsan Research Center, Helsinki, Finland; 4 Abdominal Center, Nephrology, University of Helsinki and Helsinki University Hospital, Helsinki, Finland; 5 Research Program for Clinical and Molecular Metabolism, Faculty of Medicine, University of Helsinki, Helsinki, Finland; 6 Medical Microbiology and Immunology, Unit of Biomedicine, University of Oulu, Oulu, Finland; 7 Medical Research Center, Nordlab Oulu University Hospital and University of Oulu, Oulu, Finland; 8 Department of Gastrointestinal Microbiology, German Institute of Human Nutrition Potsdam-Rehbruecke, Nuthetal, Germany; 9 Department of Diabetes, Central Clinical School, Monash University, Melbourne, VIC Australia; 10 University of Copenhagen, Copenhagen, Denmark

**Keywords:** Predictive markers, Diabetes complications, Predictive markers, Dysbiosis

## Abstract

Gastrointestinal dysbiosis is common among persons with type 1 diabetes (T1D), but its potential impact on diabetic nephropathy (DN) remains obscure. We examined whether faecal biomarkers, previously associated with low-grade gastrointestinal inflammation, differ between healthy controls and T1D subjects with and without DN. Faecal samples were analyzed for levels of calprotectin, intestinal alkaline phosphatase (IAP), short-chain fatty acids (SCFA) and immunoglobulins in subjects with T1D (n = 159) and healthy controls (NDC; n = 50). The subjects with T1D were stratified based on albuminuria: normoalbuminuria (< 30 mg/g; n = 49), microalbuminuria (30–299 mg/g; n = 50) and macroalbuminuria (≥ 300 mg/g; n = 60). aecal calprotectin, IAP and immunoglobulin levels did not differ between the T1D albuminuria groups. However, when subjects were stratified based on faecal calprotectin cut-off level (50 µg/g), macroalbuminuric T1D subjects exceeded the threshold more frequently than NDC (*p* = 0.02). Concentrations of faecal propionate and butyrate were lower in T1D subjects compared with NDC (*p* = 0.04 and *p* = 0.03, respectively). Among T1D subjects, levels of branched SCFA (BCFA) correlated positively with current albuminuria level (isobutyrate, *p* = 0.03; isovalerate, *p* = 0.005). In our study cohort, fatty acid metabolism seemed to be altered among T1D subjects and those with albuminuria compared to NDC. This may reflect gastrointestinal imbalances associated with T1D and renal complications.

## Introduction

The human gut microbiota is shaped throughout life by genetic and environmental factors^1^. Gut bacteria metabolize dietary nutrients, harvest energy from the diet, and produce essential vitamins for the need of the host. The human gut environment contributes to important functions of the immune system, and even a short-term use of antibiotics may change the balance between beneficial and pathogenic bacteria^2, 3^. Over the past decade, alterations in gut microbiota composition, known as gut dysbiosis, have been linked to a wide range of diseases such as inflammatory bowel disease (IBD), cancer, atherosclerosis, diabetes, obesity, liver diseases, psychological disorders, infections, and autoimmune diseases^4–6^.

IBD and celiac disease are multifactorial autoimmune diseases affecting the small intestine and colon. Subjects with such chronic gastrointestinal conditions are susceptible to extra-intestinal tissue damage associated with other vital internal organs such as liver, pancreas, lungs, and kidneys^7^. Subjects with IBD, celiac disease and type 1 diabetes (T1D) share many genetic and phenotypic features^8^. In T1D, the incidence and severity of gastrointestinal symptoms seem to correlate with the progression of diabetic complications, e.g. neuropathy^9^. However, due to the lack of systematic screenings, the prevalence of gastrointestinal disorders and their potential impact on the development of late diabetic complications remains obscure. We have recently shown that subjects with T1D exhibit aberrant profiles of gut microbiota and plasma metabolites compared to healthy controls. Moreover, T1D subjects with different albuminuria levels show different gut microbiota and plasma metabolite profiles^10^.

Based on this background, we analyzed a set of gastrointestinal biomarkers^10^ previously associated with intestinal inflammation (calprotectin), gut metabolite production (short-chain fatty acids), detoxification of bacterial endotoxins (intestinal alkaline phosphatase), and host defense (immunoglobulins) to investigate whether these faecal markers of gut metabolism are associated with albuminuria in a Danish cohort of adult T1D subjects with and without diabetic nephropathy.

## Materials and methods

### Study population

Between April 2016 and December 2017 subjects with T1D were recruited to participate in a cross-sectional study from the outpatient clinic at Steno Diabetes Center Copenhagen (SDCC) in Denmark. With the cohort, we aimed to perform a detailed phenotyping of T1D subjects with or without renal complications, including subjects of more than 18 years old and diagnosed with T1D according to the 2006 WHO criteria. Based on the highest historical or the present level of albuminuria, subjects with T1D were stratified into subgroups of normoalbuminuria (< 30 mg/24-h or 30 mg/g creatinine), microalbuminuria (30–299 mg/24-h or mg/g creatinine) or macroalbuminuria (≥ 300 mg/24-h or 300 mg/g creatinine). The historical albuminuria was based on two out of three consecutive urine samples collected within 1 year. Subjects with normoalbuminuria did not have any history of micro- or macroalbuminuria prior to or at enrolment. In total, 161 subjects with T1D and 50 healthy non-diabetic controls (NDC) were included. One faecal sample was missing, and one sample did not have sufficient material for analyses leaving 159 T1D faecal samples for analyses. The control group was recruited via a newspaper advertisement distributed within the area of the Copenhagen region, Denmark. The exclusion criteria for all participants were: (1) non-diabetic kidney disease; (2) renal failure (eGFR < 15 ml/min/1.73 m^2^), dialysis or kidney transplantation; (3) change in renin–angiotensin aldosterone system blocking treatment during the last month; (4) treatment with systemic antibiotics during the last two months; (5) treatment with systemic immunosuppressive treatment. Subjects were matched for age and sex between groups.

### Clinical and laboratory data

An enzyme immunoassay was used to measure the urinary albumin creatinine ratio (UACR) in three consecutive morning urine samples. The three urine samples were used to calculate the geometric mean of UACR for each subject, which is referred to as the current albuminuria level. High performance liquid chromatography was used to measure HbA_1C_. Plasma creatinine and cholesterol were measured by an enzymatic method (Vitros 5600, Ortho Clinical Diagnostics, USA) and eGFR was calculated using the chronic kidney disease-epidemiology collaboration (CKD-EPI) equation^11^. High-sensitivity C-reactive protein (hsCRP) was measured by a latex-enhanced turbidimetric immunoassay method (Cobas 8000 modul c502, Roche Diagnostics, USA). Body weight (to nearest 0.5 kg) and height (to nearest 0.5 cm) of each subject were measured and used to calculate body mass index (body weight/height^2^ [kg/m^2^]). Twenty four-hour blood pressure (24-h BP) was recorded by an oscillometric device using an appropriately sized cuff placed on the upper arm and programmed to measure blood pressure every 15 min between 7 am and 10 pm and every 30 min between 10 pm and 7 am (Takeda, TM2430, Japan)^12^. Detailed medical history was obtained including current medication and history of diabetes complications, and the information was cross-referenced with electronic patient records. Retinopathy status was acquired from retinal images taken regularly (approximately every 1–2 years) at the outpatient clinic at SDCC. Retinopathy was graded as nil, presence of or historical non-proliferative or proliferative, based on the worst eye. Peripheral neuropathy status was defined according to the latest registered vibrations perception measured by biothesiometry. Neuropathy was defined as present, when the mean vibration perception of the left and the right foot was above the age, height and sex-adjusted threshold^13^. Subjects completed a questionnaire on health and lifestyle, including information on smoking, alcohol consumption, physical activity, bowel movement and stool consistency according to the Bristol stool scale^14^. Current smokers were defined as smoking one or more cigarettes/cigars/pipes per day.

### Faecal sample collection

Subjects were instructed to collect a faecal sample at home no later than 2 weeks after the visit, following a standardized procedure including antiseptic handling, collection in sterile tubes and immediately freezing the sample in the subjects’ freezers at − 18 °C. No later than 72 h after delivery the frozen samples were transferred to the laboratory on dry ice and stored at − 80 °C until further analysis in Finland.

### Faecal calprotectin

Faecal calprotectin concentrations were determined with the human calprotectin enzyme-linked immunosorbent (ELISA) assay according to manufacturer’s instructions (Bühlmann Laboratories, Switzerland). The assay relies on tetramethylbenzidine substrate and anti-calprotectin antibodies conjugated to horseradish peroxidase. Faecal samples, for which one measurement was missing for calprotectin (n = 208) (50–100 mg), were homogenized at + 4 °C and centrifuged at 2842*g* (5200 rpm) for 5 min. The faecal extractions were diluted 1:50 for the ELISA assay. Finally, the absorbances were read at 450 nm using Synergy H1 Microplate Reader (BioTek, Winooski, VT, USA). According to the manufacturer’s guidelines, the faecal calprotectin concentrations were categorized as “no inflammation” (< 50 μg/g), “moderate inflammation” (50–200 μg/g), and “severe inflammation” (> 200 μg/g). This cut-off classification has been validated for monitoring and evaluating disease status of IBD^15^.

### Faecal short chain fatty acids

Faecal short chain fatty acids (SCFA) were measured with an HP 5890 series gas chromatograph (Hewlett-Packard, Waldbronn, Germany) equipped with an HP-FFAP column and a flame ionization detector as described earlier^16^. Briefly, 300 mg of fresh stool samples were diluted 1:5 in water and centrifuged 15000*g* for 5 min. A volume of 23.6 µl 12 mM isobutyric acid (as an internal standard), 280 µl 0.36 M HClO_4_, and 270 µl 1 M NaOH were added to 50 µl of the supernatant. The mixture was lyophilized, and the residue was re-dissolved in a mixture of 400 µl acetone and 100 µl 5 M formic acid. After centrifugation (14,000*g* for 5 min), 1 µl of the supernatant was injected into the gas chromatograph. Authentic standards were used in all runs in order to determine levels of faecal C2, C3, C4, C5, iC4 and iC5. The ratios between SCFA (sum of C2, C3, C4 and C5 concentrations) and BCFA (sum of iC4 and iC5 concentrations) were calculated. Eighteen percent (37/209) of the study subjects could not provide a sufficient amount of faecal material for the SCFA analysis. Therefore, the final number of SCFA samples was 172.

### Faecal intestinal alkaline phosphatase activity

Faecal intestinal alkaline phosphatase (IAP) activity was measured by an in-house colorimetric assay as described earlier^17^. Briefly, faecal samples (50 mg) were homogenized in 500 μl extraction buffer (0.1 mM ZnCl_2_, 1 mM MgCl_2_, 10 mM Tris–HCl pH 8.0) containing sodium calcium edetate (EDTA)-free protease inhibitor cocktail (1:50) (Roche Diagnostics, Germany), and 0.1 mm glass beads (Precellys, Bergin Technologies, Montigny, France). After homogenization, samples were centrifuged at 11,150*g* (13,000 rpm) for 10 min at + 4 °C. Supernatants were subjected to determinations of faecal IAP activity and total protein concentrations. IAP assay standards were prepared using serial dilutions of p-Nitrophenyl Phosphate (pNPP) (Sigma-Aldrich, St. Louis, MO, USA) and 1:500 diluted calf intestinal alkaline phosphatase (10,000 U/ml). Sample reactions (100 μl) constituted of 10 μl diluted (1:40–1:200) faecal supernatant, 45 μl assay buffer (0.1 mM ZnCl_2_, 1 mM MgCl_2_, 10 mM Tris–HCl pH 10.0), and 45 μl 4.56 mM pNPP stock. Standards and samples were incubated at + 37 °C for 30 min, where after the reactions were stopped by adding 20 μl 3 M NaOH. Absorbances (405/630 nm) were measured with Synergy H1 Microplate Reader. The IAP activities were calculated using the formula IAP activity (U/ml) = A/V/T, where A is the amount of pNP generated in μmol, V is the sample volume in ml and T is the reaction time in minutes. The final IAP activity levels were normalized with the faecal protein concentrations determined by the Lowry method (DC protein assay, Bio-Rad, Hercules, CA, USA).

### Faecal immunoglobulins

The levels of total IgA, IgG and IgM were determined from the faecal extracts by a chemiluminescence immunoassay as described earlier^17, 18^. Briefly, supernatants were incubated for 1 h at room temperature, and the amount of antibody bound was detected with alkaline phosphatase-labelled goat anti-human secondary antibodies for IgA, IgG and IgM (Sigma-Aldrich) using LumiPhos 530 (Lumigen, Southfield, MI, USA) chemiluminescence substrate. The luminescence readings (Victor3 multilabel counter, PerkinElmer, Waltham, MA, USA) were expressed as relative light units/100 ms.

### Statistics

Data are presented as mean (± SD) for normally distributed and as median [interquartile range] for non-normally distributed continuous variables (Tables 1, 2). All non-normally distributed variables were log-transformed before analyses. Clinical characteristics were compared across groups using unpaired Students’ t test or ANOVA for continuous variables and the χ^2^ test for categorical variables. The χ^2^ test was also used to examine the differences between faecal calprotectin-negative and -positive groups (cut-off level < 50 µg/g or ≥ 50 µg/g). Differences in the measured clinical variables between faecal calprotectin of below and above 50 µg/g were tested with Mann–Whitney U test. To test for association of neutrophils and calprotectin, Spearman’s rank correlation was used. ANOVA and ANCOVA were applied to assess for differences in the levels of calprotectin, SCFA, IAP activity and immunoglobulins between subjects with T1D and controls. These models were also applied to test for differences among the historical groups of albuminuria (normo-, micro- and macroalbuminuria). Unadjusted and adjusted linear regression models were applied to evaluate associations between current UACR level analyzed as continuous variable and the faecal biomarkers. All adjusted analyses included age, sex, 24-h systolic BP and eGFR. Two-sided *p* values less than 0.05 were considered as statistically significant. The statistical analyses were performed in R Studio version 3.4.1 (R Foundation, Vienna, Austria), SAS version 9.4 (Cary, NC, USA) or SPSS version 25.0 (Chicago, IL, USA).

**Table 1:** Clinical characteristics of the study subjects at the current visit.

	Controls	All subjects with diabetes	Normo-albuminuria	Micro-albuminuria	Macro-albuminuria	*p* value: controls vs. normo-albuminuria	*p* value: normo- vs. micro- vs. macro-albuminuria
Subjects (N)	50	159	49	50	60		
Age (years)	59 ± 13	61 ± 10	60 ± 11	62 ± 9	60 ± 10	0.642	0.652
Female (%)	22 (44)	65 (41)	20 (41)	22 (44)	23 (38)	0.907	0.834
Body mass index (kg/m^2^)	24 ± 3.2	26 ± 4.4	25 ± 4.1	26 ± 4.8	27 ± 4.1	0.258	0.192
24 h systolic blood pressure (mmHg)	133 ± 12	137 ± 12	133 ± 10	139 ± 13	139 ± 13	0.920	**0.014**
24 h diastolic blood pressure (mmHg)	80 ± 7	77 ± 6	77 ± 6	76 ± 7	76 ± 6	0.065	0.702
eGFR (ml/min/1.73 m^2^)	89 ± 14	75 ± 25	90 ± 16	81 ± 23	59 ± 23	0.727	** < 0.001**
UACR (mg/g)	4 (3–5)	14 (5–99)	3 (2–5)	12 (5–33)	152 (53–496)	0.609	** < 0.001**
Current UACR category^d^	–	–	100% normo	70% normo 30% micro	20% normo 45% micro 35% macro	**–**	**–**
HbA_1c_ (mmol/mol)	36 ± 2.7	61 ± 10.2	60 ± 8.6	60 ± 6.6	64 ± 13.0	** < 0.001**	**0.034**
HbA_1c_ (%)	5.4 ± 0.3	7.8 ± 0.9	7.6 ± 0.8	7.7 ± 0.6	8.0 ± 1.2	** < 0.001**	**0.034**
P-LDL cholesterol (mmol/l)	3.2 ± 0.8	2.2 ± 0.7	2.2 ± 0.6	2.1 ± 0.6	2.1 ± 0.8	** < 0.001**	0.583
ALT (U/l)	31 ± 7	35 ± 10	33 ± 9	36 ± 11	35 ± 9	0.200	0.283
hsCRP (mg/l)	0.8 (0.5–1.4)	1.6 (0.9–3.2)	1.1 (0.7–2.4)	1.6 (1.1–3.2)	2.1 (1.1–3.4)	**0.048**	**0.005**
Neutrophil count	3.2 (3–4)	3.8 (3–5)	3.3 (3–4)	4.0 (3–5)	4.3 (3–6)	**0.039**	**0.003**
**Smoking (%)**						0.455	0.212
Current	4 (8)	23 (15)	7 (15)	8 (16)	8 (14)		
Previous	23 (46)	68 (45)	17 (37)	19 (39)	32 (57)		
Never	23 (46)	60 (40)	22 (48)	22 (45)	16 (29)		
Alcohol (beverages/week)	7 (3–14)	7 (3–14)	7 (4–15)	7 (5–14)	7 (2–12)	0.596	0.314
Bristol stool scale^a^	4 (3–4)	3 (3–4)	3 (3–4)	3 (2.25–3)	3 (2–3.5)	0.487	0.065
Bowel movement frequency^b^	2 (1–2)	2 (2–2)	2 (2–2)	2 (2–2)	2 (1–2)	0.459	0.499
**Diabetes characteristics**							
Diabetes duration (years)	–	42 ± 15	34 ± 15	46 ± 14	44 ± 13	–	** < 0.001**
History of CD (%)	–	47 (23)	8 (16)	10 (20)	29 (48)	–	** < 0.001**
Retinopathy^c^ (%)	–	22/36/41	46/45/6	18/32/50	5/32/62	–	** < 0.001**
Neuropathy (%)	–	51 (32)	6 (12)	13 (26)	32 (53)	–	** < 0.001**

Data are shown as *n* (%), mean ± standard deviation or as median (interquartile range).

*ALT* alanine transaminase, *hsCRP* high-sensitivity C-reactive protein, *CD* cardiovascular disease. *p* values were calculated with unpaired Students’ *t* test or analysis of covariance (ANCOVA covariates were age, sex, 24-h systolic blood pressure and eGFR) for continuous variables and the χ^2^ test for categorical variables.

^a^Bristol stool scale from 1 to 7.

^b^Bowel movement frequency 1–4; 1 = Twice daily or more, 2 = Once daily, 3 = Every other day and 4 = Less than every other day.

^c^No/non-proliferative/proliferative. *eGFR* estimated glomerular filtration rate, *UACR* current urinary albumin creatinine rate.

^d^Based on results from the three albuminuria measurements at study visit. Albuminuria values (e.g. albuminuria category) may be reduced compared to the historic albuminuria measurements due to kidney protective treatment.

**Table 2:** Fecal biomarkers in (A) subjects with T1D and controls and (B) per historical albuminuria categories (normo-, micro- and macroalbuminuria).

(A) Biomarker	Controls		All T1D subjects		p value: ANOVA	p value: ANCOVA					
	Value	N	Value	N							
Calprotectin (µg/g)	6.0 (2.0–37)	50	11 (0–23)	158	NS	NS					
IAP (U/l)	71 (46–172)	50	65 (34–183)	159	NS	NS					
Fecal protein (mg/ml)	2.2 (1.7–3.9)	50	2.4 (1.8–3.0)	159	NS	NS					
Acetate (C2) (µmol/g FM)	44 (40–51)	40	39 (30–49)	132	NS	NS					
Propionate (C3) (µmol/g FM)	11 (9.0–15)	40	9 (7.0–12)	132	**0.02**	**0.04**					
Butyrate (C4) (µmol/g FM)	11 (8.1–16)	40	7.8 (4.7–12)	132	**0.01**	**0.03**					
Valerate (C5) (µmol/g FM)	1.9 (1.3–2.3)	40	1.8 (1.2–2.4)	132	NS	NS					
Iso-butyrate (iC4) (µmol/g FM)	1.5 (1.2–2.0)	40	1.7 (1.3–2.1)	132	NS	NS					
Iso-valerate (iC5) (µmol/g FM)	2.2 (1.7–2.9)	40	2.6 (1.9–3.5)	132	**0.03**	NS					
SCFA/BCFA (µmol/g FM)	16 (10–17)	40	13 (12–26)	132	**0.001**	NS					
IgA (µg/ml)	2.6 (0.8–7.3)	50	2.6 (0.9–7.8)	157	NS	NS					
IgG (ng/ml)	33 (12–74)	49	32 (15–79)	149	NS	NS					
IgM (µg/ml)	0.4 (0.1–0.8)	49	0.3 (0.1–0.3)	151	NS	NS					

(B) Biomarker	Normo-albuminuria		Micro-albuminuria		Macro-albuminuria		*p* value: normo- vs. micro-albuminuria	*p* value: micro vs. macro-albuminuria	*p* value: normo vs. macro-albuminuria	*p* value: ANOVA	p value: ANCOVA
	Value	N	Value	N	Value	N					
Calprotectin (µg/g)	14 (3.7–39)	49	11 (0–34)	50	8.4 (2.0–60)	59	NS	NS	NS	NS	NS
IAP (U/l)	64 (39–238)	49	63 (32–153)	50	74 (37–231)	60	NS	NS	NS	NS	NS
Fecal protein (mg/ml)	2.5 (1.8–3.3)	49	2.6 (2.0–3.1)	50	2.2 (1.7–2.9)	60	NS	NS	NS	NS	NS
Acetate (C2) (µmol/g FM)	38 (27–49)	40	44 (32–55)	42	39 (30–45)	50	NS	NS	NS	NS	NS
Propionate (C3) (µmol/g FM)	9.6 (6.0–12.6)	40	9.7 (6.8–12.4)	42	9.1 (6.8–11.8)	50	NS	NS	NS	NS	NS
Butyrate (C4) (µmol/g FM)	7.5 (4.5–14.5)	40	9.0 (5.6–12.0)	42	7.0 (4.7–11.0)	50	NS	NS	NS	NS	NS
Valerate (C5) (µmol/g FM)	1.6 (1.1–2.0)	40	2.0 (.5–2.6)	42	1.8 (1.3–2.4)	50	NS	NS	NS	NS	NS
Iso-butyrate (iC4) (µmol/g FM)	**1.5 (1.1–1.9)**	40	**2.0 (1.5–3.0)**	42	**1.7 (1.2–2.4)**	50	**0.02**	NS	NS	**0.05**	NS
Iso-valerate (iC5) (µmol/g FM)	**2.3 (1.7–2.7)**	40	**3.0 (2.2–3.6)**	42	**2.7 (1.9–3.8)**	50	**0.03**	NS	**0.05**	NS	NS
SCFA/BCFA (µmol/g FM)	13.3 (11–19)	40	12.0 (9.9–17.4)	42	12.2 (9.6–16)	50	NS	NS	NS	NS	NS
IgA (µg/ml)	2.2 (0.8–8.9)	49	3.6 (0.9–7.3)	49	2.8 (0.9–8.4)	59	NS	NS	NS	NS	NS
IgG (ng/ml)	38 (18–61)	44	26 (8.0–66)	49	33 (13–108)	56	NS	NS	NS	NS	NS
IgM (µg/ml)	0.2 (0.1–0.7)	47	0.3 (0.1–0.8)	46	0.3 (0.1–1.4)	58	NS	NS	NS	NS	NS

Data is shown as median (interquartile range). Log_10_-transformed values were used for all biomarkers. *SCFA* the sum of short chain fatty acids C2, C3, C4 and C5, *BCFA* the sum of branched short chain fatty acids iC4 and iC5, *IAP* intestinal alkaline phosphatase, *Ig* immunoglobulin, *FM* fecal mass. Significant differences between groups are shown in bold. *p* values were calculated by unpaired Students’ *t* test for group-wise differences (Table b), ANOVA and ANCOVA (ANCOVA covariates were age, sex, 24-h systolic blood pressure and eGFR).

### Ethics declaration

The study was conducted in accordance with the Declaration of Helsinki and was approved by the Ethics Committee of the Danish Capital Region (protocol H-15018107). All subjects gave written informed consent.

## Results

### Clinical findings

Characteristics of the 209 participants are shown in Table 1. The total cohort included 87 (42%) females and mean age was 60 (± 11) years. The subjects with T1D had a relatively long diabetes duration (mean 42 ± 15 years). Subjects with micro- and macroalbuminuria had longer duration than subjects with normoalbuminuria. The subjects with normoalbuminuria had higher hsCRP and lower LDL levels than the controls. Across the subjects with T1D the 24-h systolic BP, HbA_1C_, hsCRP, neutrophils and UACR (as expected) were higher with increasing level of albuminuria, and the eGFR was lower. Known history of cardiovascular disease, retinopathy, and neuropathy were more frequent within increasing level of albuminuria. In the group with macroalbuminuria, 48% had known cardiovascular disease, 53% had neuropathy, non-proliferative retinopathy was present in 32% and 62% had proliferative retinopathy.

### Faecal biomarkers

#### Faecal calprotectin

**Figure 1:**
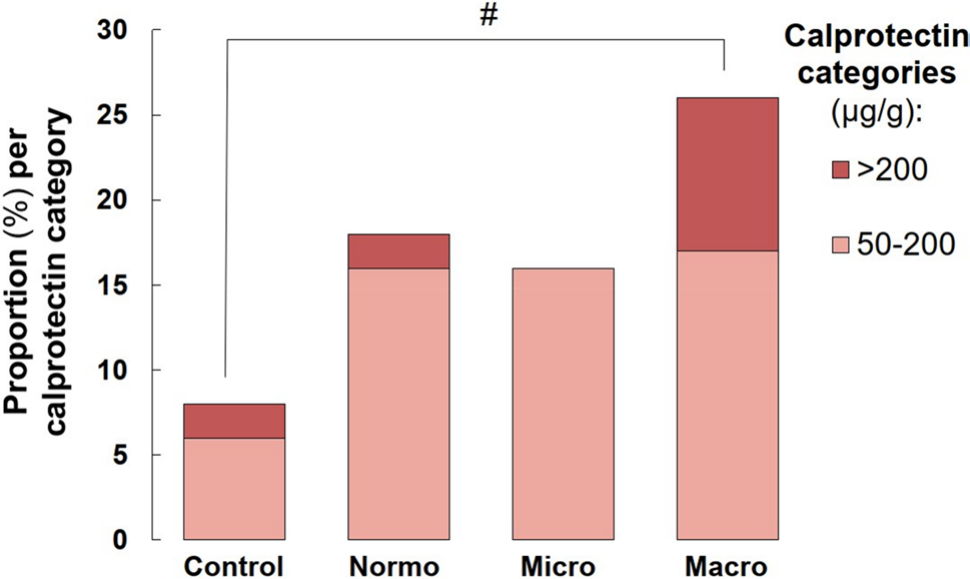
Faecal calprotectin measurements. The proportion of calprotectin positive individuals [expressing either moderate (> 50 μg/g) or high (> 200 μg/g) calprotectin levels] was higher in the macroalbuminuria group compared to controls. *Control* healthy nondiabetic controls, *Normo* T1D subjects with normal albuminuria, *Micro* T1D subjects with microalbuminuria, *Macro* T1D subjects with macroalbuminuria. ^#^p < 0.05.

Mean faecal calprotectin levels did not differ between T1D subjects and NDC or between the groups of albuminuria (Table 2). However, when subjects were stratified according to the calprotectin cut-off level for moderate to severe inflammation according to the manufacturer^15^, the percentage of subjects showing positive test results for inflammation (> 50 µg/g) were higher among subjects with diabetes and macroalbuminuria than controls: NDC 8%, T1D-normo 18%, T1D-micro 16%, T1D-macro 26% (NDC vs. T1D-macro; *p* = 0.02) (Fig. 1). No significant differences were observed in clinical phenotypes (BMI, 24-h systolic blood pressure, 24-h diastolic blood pressure, eGFR, HbA_1c_, P-LDL cholesterol, ALT, hsCRP, and blood neutrophil count), when macroalbuminuric subjects were stratified based on the negative (< 50 µ/g; n = 44) and positive (≥ 50 µ/g; n = 15) calprotectin measurements (data not shown). However, within all subjects with diabetes, we found a positive association between the faecal calprotectin levels and blood neutrophil count (r = 0.195; p = 0.015).

#### Faecal short-chain fatty acids

**Figure 2:**
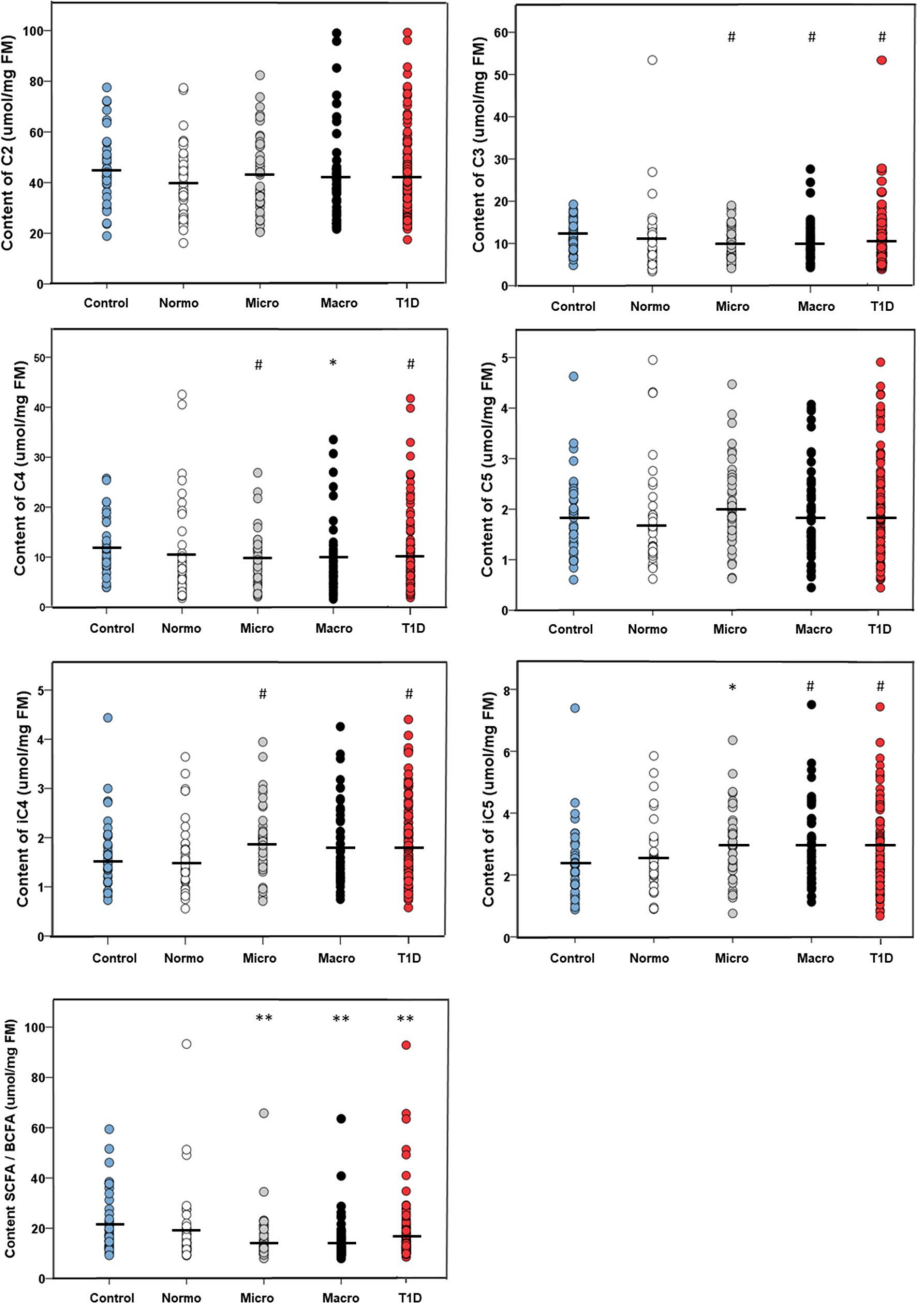
Faecal short chain fatty acids (SCFA). Acetate (C2), valerate (C5), isobutyrate (iC4) and isovalerate (iC5) levels did not differ between subjects with T1D and controls. Propionate (C3) and butyrate (C4) levels and the SCFA/BCFA -ratio were significantly lower among the subjects with diabetes compared to controls. SCFA (sum of C2, C3, C4 and C5), and branched SCFA (BCFA) (sum of iC4 and iC5). Only relevant significances between the controls (n = 40) and disease groups (Normo, n = 40; Micro, n = 42; Macro, n = 50; T1D, n = 132) are shown. ^#^*p* < 0.05; *p < 0.01; **p < 0.001.

Faecal propionate (C3) and butyrate (C4) levels were significantly lower among subjects with T1D (n = 132) than among NDC (n = 40) both in the unadjusted (C3, *p* = 0.02; C4, *p* = 0.01) and adjusted (C3, *p* = 0.04; C4, *p* = 0.03) models. Acetate (C2), valerate (C5) and isobutyrate (iC4) did not differ between the T1D and NDC groups. Isovalerate (iC5) levels were significantly higher among subjects with T1D compared to NDC in the unadjusted analysis (*p* = 0.03), but this association disappeared after adjustment. iC4 and iC5 correlated positively with each other (*p* < 0.01). The SCFA/BCFA-ratio was significantly lower among subjects with T1D than among NDC in the unadjusted model (*p* = 0.001), however, not after adjustment (Fig. 2, Table 2A).

**Figure 3:**
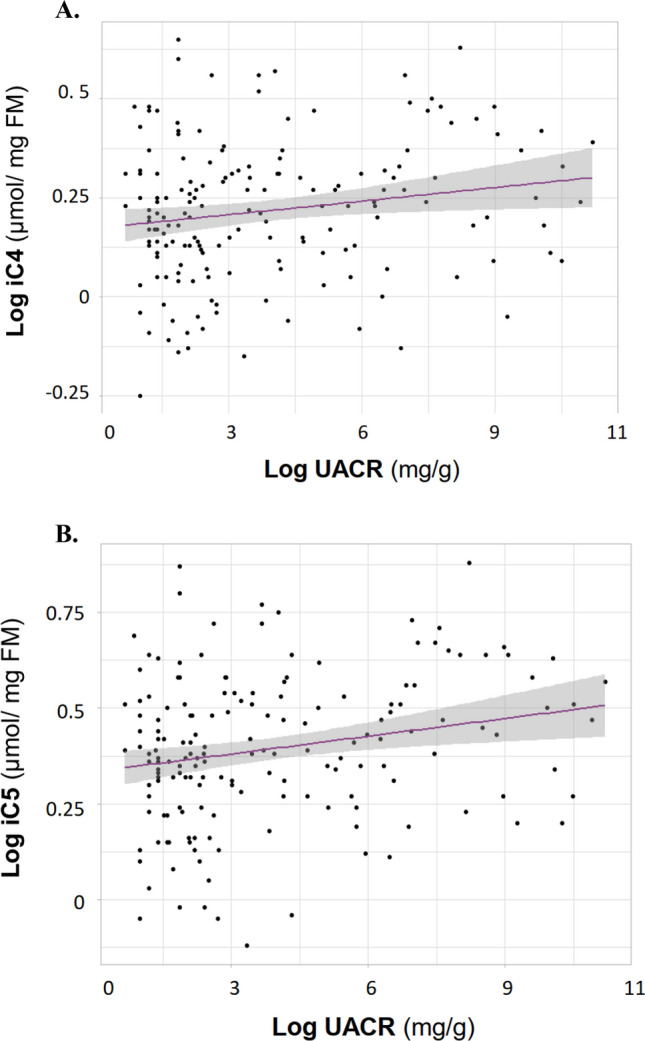
Branched short chain fatty acids (isobutyrate, iC4 and isovalerate, iC5) per urinary albumin to creatinine ratio (UACR). Both iC4 (**A**) and iC5 (**B**) correlated with UACR (iC4: R^2^ = 0.03, *p* = 0.02; iC5: R^2^ = 0.05, *p* = 0.004) in the unadjusted as well as in the adjusted model (iC4: R^2^ = 0.35, *p* = 0.03; iC5: R^2^ = 0.36, *p* = 0.005). The analyses were adjusted with age, sex, 24-h systolic blood pressure and estimated glomerular filtration rate. *FM* fecal mass. 95% CI around the regression line is shown in grey.

In the subjects with T1D, levels of C2, C3, C4, and C5 did not differ with respect to albuminuria, neither when analyzed within historical albuminuria groups (Table 2B) or as current UACR (data not shown). BCFA were higher with increasing albuminuria in unadjusted models: iC4 (normo vs. micro; *p* = 0.02), iC5 (normo vs. micro; *p* = 0.03), and iC5 (normo vs. macro; *p* = 0.05), but not after adjustment (Table 2B). Moreover, the BCFA correlated positively with current UACR in the unadjusted (iC4: R^2^ = 0.03, *p* = 0.02; iC5: R^2^ = 0.05, *p* = 0.004) as well as the adjusted (iC4: R^2^ = 0.35, *p* = 0.03; iC5: R^2^ = 0.36, *p* = 0.005) models (Fig. 3).

#### Faecal IAP activity and immunoglobulins

Faecal IAP activity and immunoglobulin (total IgA, IgG and IgM) levels did not differ between the study groups (Table 2).

## Discussion

The aim of the present study was to investigate whether faecal markers of gut metabolism are associated with albuminuria in Danish T1D subjects with and without diabetic nephropathy. We observed that mean faecal calprotectin, IAP and immunoglobulin levels did not differ between the T1D albuminuria groups. However, when the presence of moderate to severe inflammation was evaluated individuals with T1D and macroalbuminuria showed positive calprotectin levels (> 50 µg/g) three times more often compared to non-diabetic controls. Furthermore, subjects with T1D as a whole group had lower levels of the faecal propionate and butyrate compared to the healthy subjects. Taken together, our main results indicate that the T1D subjects, particularly those with macroalbuminuria, showed unfavorable changes in gut homeostasis.

Faecal calprotectin has become a valuable diagnostic tool for IBD due to its correlation with the activity and severity of the disease^19, 20^. More attention should be paid to the systematic screenings of gut related illnesses, especially in patient groups with chronic systemic diseases, as the incidence of IBD is increasing globally^21^. To date, there are very few studies, which have performed systematic screenings of IBD among subjects with T2D^22^. There is growing evidence showing that faecal calprotectin could be a useful biomarker in the assessment of intestinal health conditions in various metabolic disorders e.g. T1D, T2D, and obesity^17, 23, 24^. Gastrointestinal symptoms (e.g. early satiety, postprandial fullness, nausea, abdominal pain, vomiting, bloating, diarrhea and/or constipation) are common in subjects with diabetes^25^. In our study, higher faecal calprotectin was associated with a higher blood neutrophil count among the subjects with type 1 diabetes. One could speculate that this represents an association between intestinal inflammation (elevated levels of faecal calprotectin) and low-grade systemic inflammation (a higher number of circulating neutrophils). Increased systemic inflammation has been associated with more rapid progression of chronic kidney disease^26, 27^. In relation to the development of diabetic complications, the role of gut-related diseases remains obscure. We observed that a higher proportion of subjects with T1D and macroalbuminuria had elevated levels of faecal calprotectin compared to non-diabetic controls, which could be interpreted as an early sign of gastrointestinal inflammation. What could be possible explanations for the elevated faecal calprotectin values in subjects with macroalbuminuria? T1D subjects with micro- and macrovascular complications use more often antibiotics and have higher systemic endotoxin levels compared to those without diabetic complications^28–31^. Changes in lifestyle related factors (e.g. diet, physical activity, medication etc.) may increase the risk of gut dysbiosis, which could eventually lead to expansion of pathogenic bacteria. It is also evident that decreased intestinal barrier function in combination with leakage of gut derived microbial toxins could accelerate and aggravate cardiovascular disease and kidney impairment including elevated albuminuria. On the other hand, kidney impairment with following accumulation of toxic metabolites (e.g. uremic compounds) in chronic kidney disease, may also have deleterious effects on the gut^32^*.* Thus, pathophysiological mechanisms related to the gut-kidney axis could be bidirectional. The fact that we did not observe any significant differences among individuals with T1D and different levels of albuminuria could have different reasons including: (1) the number of study subjects were too low; and (2) the albuminuria groups were relatively well-treated as the measured median (IQR) albuminuria levels were 12 (5–33) and 152 (53–496) for the micro- and macroalbuminuria groups, respectively. We observed that the proportion of macroalbuminuric subjects with positive test result for calprotectin (> 50 µg/g) was higher compared to NDC. However, faecal calprotectin was not, as we would have expected, associated with current albuminuria levels. The lack of association between continuous calprotectin and albuminuria measures are most likely explained by the non-linear behaviour of these parameters and the small sample size.

In the present study, T1D subjects with kidney disease displayed lower faecal propionate and butyrate levels compared to NDC, which could be explained by observed changes in gut microbiota composition^10^. These results are also in line with our earlier findings—faecal propionate and butyrate levels were lower in Finnish T1D subjects compared to non-diabetic healthy controls^17^. The abundant SCFA acetate, propionate and butyrate, contribute to the regulation of glucose and lipid homeostasis^33–35^ by ligating to free fatty acid receptors (e.g. FFAR2) located on hematopoietic, stromal and intestinal epithelial cells. Their binding regulates the inflammasome, the main component of the functional innate immune system^36^. Propionate levels have previously been linked to satiety and the risk of obesity and insulin resistance^37^. High levels of propionate reduce food intake by stimulating the expression of gut hormones peptide YY (PYY) and glucagon-like peptide 1 (GLP-1)^38^. Life-style related factors, e.g. high carbohydrate intake, has been associated with decreased levels of faecal propionate and butyrate in subjects with type 2 diabetes^39^. Butyrate inhibits the activity of histone-deacetylase complex, responsible for transcription and chromatin remodeling, through which it activates regulatory T cells in immunity. It controls pathogens also directly in the intestine by reinforcing tight junctions, hereby increasing the barrier to pathogens. Previous studies have shown that butyrate-producing bacteria in the gut are less abundant in subjects with T1D^40–42^ as well as in subjects with chronic kidney disease^43, 44^. Future studies will be needed in order to uncover whether changes in dietary factors, medication, physical activity etc. will explain the observed changes in intestinal SCFA-levels in individuals with diabetic kidney disease.

Faecal concentrations of the BCFA, isobutyrate (iC4) and isovalerate (iC5), correlated positively with albuminuria in our study. BCFA originate from bacterial degradation of proteins in the colon, while the SCFA (C2–C5) originate from carbohydrate breakdown^45^. The fact that iC4 and iC5 correlated with each other could indicate that their metabolic functions are different from those of the linear SCFA (C2–C5)^46^. It has been suggested that a decrease in the SCFA/BCFA-ratio might reflect unfavorable conditions, where the local SCFA production is reduced at the expense of increased amino acid fermentation^47^. We observed that the SCFA/BCFA-ratio was significantly lower among the subjects with T1D compared to the healthy controls. It is of note that clinical trials with pre- and probiotics have shown potential therapeutic effects in the management of various metabolic diseases^48, 49^, but further evidence regarding their beneficial effects in general and in T1D is needed. In subjects with ulcerative colitis, oral supplementation of inulin-type beta-fructans for 9 weeks improved colon health and increased colonic butyrate (C4), whereas the isobutyrate (iC4) and isovalerate (iC5) levels were markedly decreased^50^. Moreover, increased faecal levels of iC4 and iC5 have been linked to unfavorable changes in the composition of serum lipids and gut microbiota in subjects with hypercholesterolemia^51^.

We examined the fecal biomarkers in relations to diabetic nephropathy both as historic albuminuria levels (divided in groups) as well as measured current level of albuminuria. The results for associations with the fecal biomarkers were consistent for both measurements, however for the BCFA results, the association with current albuminuria remained significant after adjustment. This may be explained by loss of power in the group division, however an explanation could also be the fact that the BCFA are reflective of the situation at the time, when the current albuminuria status was recorded. The historic albuminuria level reflects worst ever status. However, the historic level is modified by treatment with renin–angiotensin–aldosterone system (RAAS) inhibitors which is reflected in the current albuminuria status. We do not know whether a reduction in the albuminuria level with RAAS inhibitors improves renal histology or just perform hemodynamic modifications leading to albuminuria reductions and whether such modifications could alter other features such as BCFA.

In contrast to our previous study in Finnish subjects with T1D^17^, we did not observe differences in the IAP activity or immunoglobulin levels between Danish T1D and NDC, or within the level of albuminuria. A reason for this discrepancy may be due to differences in the two cohorts studied. The Finnish macroalbuminuric subjects had higher median albuminuria level compared to the Danish cohort (978 [231–2812] vs. 152 [53–496]) suggesting more advanced disease.

This study has also limitations. The design is cross-sectional, which does not allow to evaluate direct causality between gut related biomarkers and progression of diabetic nephropathy. The primary aim of the study was to investigate faecal biomarkers, however, future data on gut microbiome, metaproteomics, and faecal metabolomics have not been possible to include in the current analysis. Such data might have helped to create a more comprehensive picture of the gut-related phenotypes. In addition, the lacking information of nutrition or dietary habits, could have had an influence on the faecal biomarkers. Therapies targeting diabetes (inflammation, dyslipidemia, poor glucose control etc.) and kidney disease (albuminuria, blood pressure etc.) could also have beneficial effects on the gastrointestinal health, which may lead to underestimation of the effects.

## Conclusions and future directions

In the present study, we found putative associations between gut biomarkers, T1D and the presence of kidney complications. Subjects with T1D showed unfavorable intestinal SCFA levels compared to healthy controls. In the T1D group, BCFA were positively associated with albuminuria. Moreover, subjects with T1D and macroalbuminuria had more frequently elevated levels of faecal calprotectin although the mean levels were not different between the groups. These observations may reflect that gastrointestinal diseases could play a significant role also in diabetic nephropathy at an early stage. Our observations together with previous findings suggest that gastrointestinal problems related to low-grade inflammation and dysbiosis might be more common in T1D than previously anticipated. To disentangle the putative interconnections between the gut and the kidney related disorders as well as incidence and severity of gastrointestinal related symptoms in subjects with T1D, longitudinal follow-up and intervention studies are needed.
